# 新药时代原发免疫性血小板减少症个体化治疗展望

**DOI:** 10.3760/cma.j.issn.0253-2727.2021.11.017

**Published:** 2021-11

**Authors:** 琳琳 黄, 恒 梅

**Affiliations:** 华中科技大学同济医学院附属协和医院血液病研究所，武汉 430022 Institute of Hematology, Union Hospital, Tongji Medical College, Huazhong University of Science and Technology, Wuhan 430022, China

原发免疫性血小板减少症（ITP）是常见的出血性疾病，发病机制复杂且异质性强，患者往往疗效不一。随着多种新药的临床应用，根据不同患者的发病机制进行个体化诊疗成为今后ITP研究的重要方向。本文回顾ITP发病机制的最新进展并探讨该病的个体化治疗策略。

一、不同维度的ITP发病机制

（一）B细胞及自身抗体参与的发病机制

体液免疫是ITP的主要发病机制，约占60％。参与发病的自身抗原主要为血小板表面糖蛋白GPⅡb/Ⅲa和GPⅠb/Ⅸ/Ⅴ。患者CD154^+^ T细胞对血小板表面抗原失去耐受，向淋巴结及脾脏的B细胞递呈抗原，使其活化而产生自身反应性抗体[1]，介导血小板的清除。

血小板清除方式与抗体特异性相关：抗GPⅡb/Ⅲa抗体调理的血小板主要在脾脏的内皮网状系统经Fcγ受体（FcγR）依赖途径清除；抗GPⅠb/Ⅸ/Ⅴ抗体调理的血小板则发生活化和去唾液酸化，经肝细胞Ashwell-Morell受体（AMR）识别结合，被肝细胞捕获和Kupffer细胞吞噬清除[2]–[3]。后者存在一正反馈环路，即血小板活化和去唾液酸化相互促进，使抗体浓度较低患者也可能发生血小板严重减少[2]。同时，两种途径存在一定的交叉[2]：部分抗GPⅡb/Ⅲa抗体可能通过FcγRⅡa诱导血小板活化和去唾液酸化；抗GPⅠbα抗体也可以通过FcγRⅡa加重血小板清除。在小鼠体内的研究则发现，除了抗体类型，抗体浓度也会影响血小板的体内清除甚至肝脏促血小板生成素（TPO）的表达[3]，表明抗体特异性并不是影响血小板清除的唯一因素。

（二）T细胞参与的发病机制

T细胞介导的免疫调控紊乱和细胞毒性是ITP患者发病的又一重要机制，这部分患者血小板相关自身抗体表达较少。

调节性T细胞（Treg）对调节和维持免疫耐受至关重要，ITP患者体内Treg数量减少且功能受损。树突状细胞（DC）以吲哚胺2,3-双加氧酶1（IDO-1）依赖的方式促进T细胞转化为Treg[4]，CD16^+^单核细胞亚群则促进Th1表型分化，抑制IL-17^+^CD4^+^细胞增殖和Treg分化[5]。患者体内IDO-1的表达减少和活性降低[4]，以及CD16^+^单核细胞亚群的扩增[5]，使得Treg水平明显下降。同时，Treg功能受损，DC成熟增加而耐受性降低，更容易递呈自身抗原而诱发自身免疫紊乱[4]。

CD8^+^ T细胞介导的细胞毒性直接参与血小板破坏。在慢性ITP患者体内，细胞毒性T细胞被自身反应性淋巴细胞激活，通过细胞凋亡和穿孔素/颗粒酶介导的细胞毒性作用破坏血小板和（或）巨核细胞，介导发病。活动性ITP患者体内CD3^+^ T细胞表面杀伤细胞免疫球蛋白样受体（KIR）表达的降低则加重了这一过程[6]。除了细胞毒性作用，CD8^+^T细胞还可以诱导血小板去唾液酸化及其经肝脏的清除[7]。

此外，慢性ITP患者Th17细胞及相关细胞因子水平升高，Th1表型增加，共同参与疾病发生；滤泡辅助性T细胞表面表达CD154（CD40L）并分泌IL-21，刺激脾脏B细胞分化为生发中心B细胞和浆细胞，产生抗GPⅡb/Ⅲa抗体，介导T细胞依赖性体液免疫应答的发生[1]。多种T细胞亚群及相关细胞因子相互影响，诱导发病。

（三）其他细胞参与的发病机制

巨核细胞是血小板的重要来源。GPⅡb/Ⅲa和GPⅠb/Ⅸ/Ⅴ等表面糖蛋白的表达，使巨核细胞成为自身抗体的作用靶点之一，其生成、成熟及凋亡因此受抑，产板功能受损。一些自身抗体阴性的持续性或慢性ITP患者体内，则存在着独立于骨髓环境的内在巨核生成缺陷。同时，血小板生成不足的ITP患者体内TPO浓度仅正常或略有升高，且TPO受体激动剂（TPO-RA）对其有效，说明TPO表达相对不足。

一些血小板表面分子具有免疫调节作用：如血小板表面CD40L表达可以促进DC成熟，诱导B细胞同种型转换，增强CD8^+^T细胞反应并激活自身反应性淋巴细胞产生抗血小板自身抗体[8]；血小板CXCL4可以促进CD4^+^Th17细胞活性[9]等，介导血小板的破坏。

二、ITP发病机制的鉴别

ITP尚无确诊方法，但相关实验室指标和临床反应可能区分不同ITP个体发病机制，有助于筛选患者人群，提高诊治效率。

自身抗体是判断患者发病机制的重要标志，可辅助治疗选择。临床常用单克隆抗体俘获血小板抗原技术（MAIPA）来检测患者血浆中的自身抗体，此种方法灵敏度低，特异性高，可以显示特异性血小板糖蛋白自身抗体。抗GPⅠb/Ⅸ/Ⅴ抗体阳性患者发病机制更加复杂，对靶向体液免疫途径的多种疗法如糖皮质激素、静脉注射免疫球蛋白（IVIG）及利妥昔单抗等的反应率都较低，易发生多重耐药[10]。一般来说，自身抗体的特异性与相应的血小板清除机制相关，但明确血小板清除路径需要更加精准的生物标志物。血小板经肝脏清除与血小板表面糖蛋白的唾液酸化水平相关，蓖麻凝集素Ⅰ（RCA-1）染色和唾液酸神经氨酸酶-1（NEU-1）染色[2]等方法，可用于评估血小板去唾液酸化程度而推断其病理过程。

基线TPO水平可以预测患者的治疗反应，并在鉴别低增生性血小板减少症和消耗性血小板减少症方面具有诊断价值[11]。相对较高的TPO水平可能与TPO-RA治疗无反应相关，提示患者巨核细胞严重减少或实为继发性ITP[11]。同时，基线TPO水平也可为药物选择提供参考，TPO水平轻度升高（100～200 ng/L）的患者更容易对罗米司亭产生反应[12]。

ITP患者的自身免疫标志物阳性率较高，常见的自身免疫标志物并不能预测血小板自身抗体阳性，但抗甲状腺过氧化物酶抗体（anti-ThyPeroxAb）、狼疮抗凝物（LAC）、抗核抗体（ANA）及抗磷脂抗体（aPL）可能和预后相关[13]–[14]：anti-ThyPeroxAb阳性患者缓解率较低；而后三种抗体阳性的患者血栓形成风险增加，使用TPO-RA时应慎重。

此外，年龄和既往治疗反应对脾切除术有一定的预后价值：老年患者（≥65岁）复发率高且易产生术后并发症；既往糖皮质激素和IVIG治疗有效有助于证实其ITP诊断，相对于既往治疗无效的患者更有可能产生反应[15]。

三、基于不同ITP发病机制的治疗

ITP病理生理机制的阐明及相关鉴别手段的发展为其靶向治疗创造了条件，以ITP不同发病环节为靶点的二线治疗逐渐完善，临床上根据患者发病机制和既往治疗反应的不同进行特异性选择，将有助于改善患者结局。

（一）减少自身抗体介导的血小板破坏

1. 减少抗体来源：利妥昔单抗（RTX）靶向CD20^+^ B细胞，抑制抗体产生，从而诱导病情缓解。RTX耗竭B细胞后，相关细胞因子如B细胞活化因子（BAFF）等分泌过量，促进致病性浆细胞向长寿命浆细胞分化并在脾脏定居，使患者耐药[16]。这也是部分患者RTX治疗无效、脾切除有效的原因。因此，RTX与anti-BAFF联用，以防止长寿命浆细胞的产生，或许可以防止患者耐药。使用RTX治疗时，抗GPⅡb/Ⅲa抗体阳性患者自身抗体水平下降更为明显，但治疗时自身抗体的存在和治疗后自身抗体水平的下降与治疗反应并不相关，使得自身抗体对RTX治疗反应的预测价值尚有争议[10],[17]。这一现象也提示独立于自身抗体的缓解机制的存在，但有关RTX的具体作用机制仍待阐明。

2. 缩短抗体寿命：作为MHCⅠ类分子家族的一员，新生儿Fc受体（FcRn）表达于血管内皮细胞、上皮细胞、造血细胞等多种细胞表面，可保护血浆IgG免受溶酶体破坏，使其重新释放入血浆并获得较长的血清半衰期。此外，它还可以递呈抗原，启动并联系体液和细胞免疫应答。因此，抑制FcRn可以减少ITP患者自身抗体的循环水平，达到治疗效果。多种FcRn抑制剂（Efgartigimod、Rozanolixizumab等）已经完成Ⅱ期临床试验，不仅能有效提高血小板计数，还具有良好的安全性和耐受性，具有十分广阔的应用前景。

3. 阻断下游信号通路：福他替尼（Fostamatinib）是一种选择性脾酪氨酸激酶（Syk）抑制剂，此类药物可抑制FcR触发的、Syk依赖的信号转导和细胞骨架重排，从而减少巨噬细胞介导的血小板破坏。抗GPⅡb/Ⅲa抗体结合的血小板多由此清除，Syk抑制剂得以发挥保护作用。同时，Syk抑制剂还可以使B细胞发育受抑而影响抗体形成[18]，对多种自身免疫病潜在有效。

与Syk相似，表达于B细胞和多种固有免疫细胞的布鲁顿酪氨酸激酶（BTK）也是多种免疫相关信号通路的关键激酶。研究已发现，一种选择性、共价、可逆的BTK抑制剂Rilzabrutinib可以阻断B细胞活化、抑制抗体产生并中和致病抗体，有望治疗多种免疫性疾病[19]。一项在复发ITP患者中进行的开放标签Ⅰ/Ⅱ期研究[20]显示，口服Rilzabrutinib可使患者获得持久有效的血小板反应，且耐受良好。Rilzabrutinib抑制血小板破坏而不影响血小板聚集[19]–[20]，其临床效益也正在进一步探索当中，或许会在不久的将来成为一种新的治疗选择。

4. 去除破坏部位：脾脏是血小板破坏的主要部位，脾切除可有效减少血小板破坏并获得较为持久的反应。但脾切除也会带来手术并发症、感染和血栓形成等风险，应综合疾病特征、既往治疗反应、年龄和生活方式等因素做出手术决定，必要时可进行相应的预防治疗[15]。目前少有数据提供脾切除与其他二线疗法的长期疗效对比，因此很难做出基于证据的治疗选择。

（二）针对细胞免疫及免疫调控缺陷的干预

作为经典化疗药物，环孢素A选择性抑制抗原诱导的CD4^+^淋巴细胞活化和IL-2等细胞因子产生，并使B细胞生长分化间接受抑；霉酚酸酯（MMF）和硫唑嘌呤则主要通过抑制嘌呤合成来抑制T/B淋巴细胞的增殖和功能。达那唑在体内与包括糖皮质激素受体在内的多种类固醇受体结合，间接增加糖皮质激素效应而导致免疫抑制，减少血小板破坏。全反式维甲酸（ATRA）可上调TGF-β水平而促进Treg的发育和功能，诱导缓解[21]。上述药物理论上有效，但其用于ITP治疗的证据仍不充分，且不良反应较大，目前仅根据医师经验及患者情况进行个体化选择，或作为联合用药以增强疗效。

（三）促进血小板产生

血小板产生不足在部分ITP患者的发病中占主导地位，增加血小板产生将有效提高此类患者的血小板计数。TPO-RA通过与TPO受体相应位点结合，模拟内源性TPO的作用，促进巨核细胞增殖和血小板产生[22]。此外，TPO-RA可能还具有免疫调节作用，使部分患者在停药后仍可维持较长时间的缓解。TPO-RA的有效率高达60％～80％[10]。目前FDA已批准三种TPO-RA用于成人慢性ITP的治疗，最常见的为艾曲波帕和罗米司亭；阿伐曲泊帕肝毒性小、易于口服，是一种新型药物，但其长期安全性数据仍待补充。艾曲波帕与罗米司亭的疗效并无明显差异，但相较于罗米司亭，艾曲波帕治疗患者出血相关不良事件发生率和抢救药物使用率更低[23]。使用TPO-RA治疗时，一种药物治疗无效的患者更换为另一种药物仍可能有效[22]；单药治疗无效者尝试联用免疫抑制剂（如低剂量泼尼松）也可能诱导应答[12]。

（四）潜在有效的新药物和新靶点

西罗莫司通过调节ITP患者的T/B细胞亚群来促进和重建外周耐受，硼替佐米可以诱导长寿命浆细胞的凋亡[24]。两种药物都可以减少血小板相关自身抗体的产生而对ITP患者潜在有效。

低剂量地西他滨（DAC）以去甲基化作用促进巨核细胞成熟，增加血小板的外周释放，并通过调节T细胞亚群诱导持续反应。虽然其安全性和有效性在相关研究中得到了验证，但最佳治疗剂量和周期仍待进一步探索。

一些细胞表面分子对免疫细胞的活化和功能至关需要，针对这些分子的靶向药物可以抑制免疫细胞相互作用而诱导免疫抑制及免疫耐受；神经氨酸酶抑制剂磷酸奥司他韦可通过抑制血小板去唾液酸化减少血小板的外周破坏，恢复血小板计数[2]；靶向并抑制经典补体通路的药物在一项纳入慢性难治患者的临床试验中也获得了持久的疗效和良好的安全性[25]。随着研究深入，有望发现更多有价值的新靶点。

（五）联合治疗策略

指南推荐难治患者采用联合治疗，联合应用的药物作用机制应不同。硫唑嘌呤、环孢素A和霉酚酸酯分别抑制不同T细胞途径，以较低剂量联合可提高疗效并降低毒性。TPO-RA出现后，包含TPO-RA在内的多药联合方案取得了显著疗效。一项在18例慢性/持续性ITP患者中进行的回顾性研究[26]显示，TPO-RA（艾曲波帕/罗米司亭）、免疫抑制剂（环孢素A/霉酚酸酯）联合IVIG的有效率可达72％（13/18）。联合方案中TPO-RA与减少血小板破坏药物联用似乎是治疗起效的关键[27]。

随着二线药物地位的提升，糖皮质激素与各种二线药物的联合方案得到普遍应用。地塞米松同TPO-RA[28]、重组人血小板生成素（rhTPO）[29]或奥司他韦[30]的联合方案在初治人群也效果良好，有望成为新的一线疗法。不同二线药物的联合也可提高疗效：相较于达那唑单药治疗，联用ATRA可显著提高患者的长期缓解率；rhTPO联合RTX或达那唑也使难治患者显著获益 [27]。

联合方案中相对较低的药物剂量降低了治疗毒性，难治患者发病机制的复杂性也使得多药联合方案成为更好的选择。但联合治疗的适应证、药物种类或剂量、治疗周期等尚处于探索阶段。

三、结语

个体化思维是未来ITP诊疗的核心，新型二线疗法相对于传统疗法的疗效提升显示出靶向治疗的正确性。然而，目前ITP确切发病机制有待进一步探究，确诊及预后相关生物标志物仍然欠缺，ITP个体化诊疗体系亟待完善。因此，在发病机制研究不断深入、新药不断发展的同时，寻求临床实用的实验室指标，搭建从发病机制到特异性治疗的桥梁，将是ITP个体化诊疗的一大进步。
